# Association of Single Nucleotide Polymorphisms of IL23R and IL17 with Ulcerative Colitis Risk in a Chinese Han Population

**DOI:** 10.1371/journal.pone.0044380

**Published:** 2012-09-11

**Authors:** Pengli Yu, Fangcheng Shen, Xiaofei Zhang, Risheng Cao, Xiaodan Zhao, Pengfei Liu, Huiming Tu, Xiaozhong Yang, Ruihua Shi, Hongjie Zhang

**Affiliations:** 1 Department of Gastroenterology, the First Affiliated Hospital of Nanjing Medical University, Nanjing, China; 2 Department of Gastroenterology, the People's Hospital of Jiangyin, Jiangyin, China; 3 Department of Gastroenterology, the Fourth People's Hospital of Wuxi, Wuxi, China; 4 Department of Gastroenterology, the First Hospital of Huaian, Huaian, China; Sudbury Regional Hospital, Canada

## Abstract

**Background:**

Previous studies implicated that IL23R and IL17 genes play an important role in autoimmune inflammation. Genome-wide association studies have also identified multiple single nucleotide polymorphisms (SNPs) in the IL23R gene region associated with inflammatory bowel diseases. This study examined the association of IL23R and IL17A gene SNPs with ulcerative colitis susceptibility in a population in China.

**Methodology:**

A total of 270 ulcerative colitis and 268 healthy controls were recruited for the analyses of 23 SNPs in the IL23R and IL17A regions. Genomic DNA was extracted and analysis of these 23 SNPs using ligase detection reaction allelic (LDR) technology. Genotype and allele associations were calculated using SPSS 13.0 software package.

**Principal Findings:**

Compared to the healthy controls, the variant alleles IL23R rs7530511, and rs11805303 showed a statistically significant difference for ulcerative colitis susceptibility (0.7% *vs* 3.3%, *P* = 0.002; 60.4% *vs* 53.2%, *P* = 0.0017, respectively). The linkage disequilibrium (LD) patterns of these SNPs were measured and three LD blocks from the SNPs of IL23R and one block from those of IL17A were identified. A novel association with ulcerative colitis susceptibility occurred in haplotypes of IL23R (Block1 H3 *P* = 0.02; Block2 H2 *P* = 0.019; Block3 H4 *P* = 0.029) and IL17A (H4 *P* = 0.034). Pair-wise analyses showed an interaction between the risk haplotypes in IL23R and IL17A (*P* = 0.014).

**Conclusions:**

Our study demonstrated that rs7530511, and rs11805303 of IL23R were significantly associated with ulcerative colitis susceptibility in the Chinese population. The most noticeable finding was the linkage of IL23R and IL17A gene region to ulcerative colitis risk due to the gene-gene interaction.

## Introduction

Ulcerative colitis (UC) is characterized by chronic and relapsing inflammation in the gastrointestinal tract, and mostly affects the colorectal region of the gastrointestinal tract [1]. As one of two major phenotypes of inflammatory bowel disease (IBD), UC still remains of unknown etiology. Several studies have indicated that UC is caused by complex interactions of environmental, genetic, and immunoregulatory factors. Of these, immune dysregulation and genetic factors are thought to play important roles in the pathogenesis of UC [2]–[3]. Genome-wide association studies and linkage analyses have uncovered some susceptibility genes related to IBD, such as nucleotide-binding oligomerization domains 2 (NOD2), autophagy-related gene 16 like 1 (ATG16L1), and IL-23 receptors (IL23R) [3]–[7]. Furthermore, several meta-analyses of genome-wide association scanning studies showed some susceptibility genes related to IBD [8]–[9]. However, the genetic polymorphisms located within these genes did not thoroughly explain the pathogenesis of IBD or the variations in disease phenotypes.

Recently, it is shown that the IL-23/IL-17 axis plays an important role in IBD development [10]. IL-23 is essential for expansion and maintenance of Th17 response [11]–[12]. Moreover, IL-23R was expressed on a variety of cells and can directly activate a subset of macrophages, monocytes, natural killer cells, and dendritic cells which secrete IL-17[13]–[14]. As for IL23R polymorphisms, a study by Lappalainen *et al*. [15] demonstrated that IL23R polymorphisms (rs1004819, 10889677, rs2201841, rs11465804, and rs11209026) increased Crohn’s disease (CD) susceptibility in a Finnish population, while another study revealed that an association of IL23R with UC, but not with CD in Swedish patients [16]. A study from Magyaril *et al*. [17] indicated that the IL23R gene variants rs10889677 C/A and rs2201841 T/C appeared to increase the susceptibility of CD but not of UC in a Hungarian population. These results showed that functional variants in the IL23R gene were identified as susceptibility loci in Caucasian IBD patients; however, literature on genetic associations of IBD in Asian populations is currently limited [18]. Therefore, further study on the association of IL23R polymorphisms with UC definitely needs to be performed to identify functional variations in IL23R in Asian patients with UC. In addition, accumulated evidence has demonstrated that IL-17 as the downstream cytokines of IL-23 plays an important role in IBD development. Others have shown that IL17A or IL17F gene polymorphisms play a role in Rheumatoid Arthritis (RA) and asthma [19]–[22], but there are fewer reports on associations of the IL17A gene polymorphisms with UC; particularly, there are no reports about IL17A gene polymorphism in Chinese UC patients.

Thus, in this study, we examined the distribution of 16 SNPs in the IL23R gene and 7 SNPs in the IL17A gene for analysis of the allele/genotype–phenotype association and gene-gene interaction, which could provide insightful information for their role in UC development in a Chinese Han population.

## Methods

### Study Population

In this study, we recruited 270 cases of UC and 268 healthy controls from four different hospitals (The First Affiliated Hospital of Nanjing Medical University, the First Hospital of Huaian, the Fourth People's Hospital of Wuxi, and the People's Hospital of Jiangyin) between 2008 and 2011. The diagnosis of UC was based on standard clinical, endoscopic, radiological, and histological criteria [23]. Cases were determined according to Montreal classification system, and cases with indeterminate colitis were excluded. The UC lesions were classified according to the Montreal classification [24], and severity was assessed by the Mayo disease activity index [25]. Healthy controls consisted of a randomly selected and ethnically matched healthy individuals without tumors, autoimmune diseases, and IBD family history. The main clinical characteristics of the study population are summarized in Table 1. In addition, the SNP positions of IL23R and IL17A in genomic DNA are listed in Table S1.

**Table 1:** The basic characteristics of the UC patients.

Clinical Characteristics	UC (n = 270)
Mean age ± SD (range)	44.49±14.24
(age of onset)	(14–77)
Male	48.1%
Female	51.9%
Lesion localization	
ulcerative proctitis	17.8%
left sided UC	43.0%
extensive UC	39.2%
Perianal lesions	
anal fistula	1.9%
anal fissure	3.7%
perianal abscess	1.5%
Extraintestinal manifestations	
Aphthous ulcer	2.9%
Arthritis/Arthragia	9.6%
IBD-related skin diseases	1.5%
Hepatopathy	1.9%
Oculopathy	0
Clinical severity	
Mild	39.3%
Moderate	51.9%
Severe	7.4%
Remission	1.4%
Endoscopic severity	REI 4.52±2.279
Family history	0.7%
Smoking	
Never smoking	80.0%
Quit smoking	5.9%
On smoking	14.1%
Appendectomy	2.2%

Note: REI Rachmilewitz endoscopic index. UC lesions were classified according to the Montreal classification. UC disease severity was assessed by the Mayo disease activity index. The control group included 136 males and 132 females, and the average age was 47.1±12.9.

### Ethical Approvals

This study was approved by the ethics committees of all four hospitals (Ethics Committee of the First Affiliated Hospital of Nanjing Medical University, Ethics Committee of Jiangyin People's Hospital, Ethics Committee of the Fourth People's Hospital of Wuxi, and Ethics Committee of the First Hospital of Huaian) involved in this study, and all patients signed informed consent forms for participation in this study. Clinical data were collected from the patients’ medical history and from questionnaires.

### Genomic DNA Extraction and Genotyping Analysis

Approximately 2 ml of blood from the cases and controls was collected and stored in tubes containing EDTA. Blood cells and serum were separated in conditions of 4°C at 3000 rpm/min for 10 min within 4 h of blood collection. Genomic DNA was then extracted from peripheral blood lymphocytes using the RelaxGene Blood DNA System (TianGen Biotech, Beijing, China). The single nucleotide polymorphisms were genotyped by a predesigned ligase detection reaction allelic (LDR) technology (Shanghai Jierui Bio Co., Ltd., Shanghai, China). The PCR amplification was carried out in a volume of 15 µl, including 1 µl of genomic DNA, 1.5 µl 10× buffer, 1.5 µl MgCl_2_, 0.3 µl dNTP (10 mM), 0.5 µl of each primer and H_2_O for an initial DNA denature at 94°C for 2 min and then 35 cycles of 94°C for 20 s, 56°C for 20 s, and 72°C for 40 s and a final extension at 72°C for 3 min. Then, 3 µl of the PCR product was mixed with 1 µl of 10× Taq DNA ligase buffer, 0.125 µl of 40 U/µl Taq DNA ligase, 0.01 µl of probe (10 p)/Article and then PCR amplified for 30 cycles of 94°C for 30 s and 56°C for 3 min. After that, 1 µl of PCR product was added to 10 µl of sample loading buffer containing a molecular weight marker, and then denatured at 95°C for 3 min and immediately put into an ice bath for DNA sequence analysis using an ABI 3730 system.

**Table 2:** Allele frequency variants in IL23R and IL17A genes.

		Variant	Controls	UC		
SNP		allele	n (%)	n (%)	OR+	95% CI+
IL23R	rs1004819	T	306 (57.1)	334 (61.9)	1.219	(0.955–1.555)
	rs1495965	A	270 (50.4)	264 (48.9)	0.942	(0.742–1.197)
	rs1884444	T	190 (35.4)	160 (29.6)	0.767	(0.594–0.990)
	rs2201841	C	382 (71.3)	408 (75.6)	1.246	(0.950–1.634)
	rs6677188	A	114 (21.3)	96 (17.8)	0.800	(0.591–1.083)
	rs7517847	G	226 (42.2)	204 (37.8)	0.833	(0.652–1.063)
	rs7530511	T	18 (3.3)	4 (0.7)	0.215	**(0.072–0.639)** *
	rs10489629	A	400 (74.6)	412 (76.3)	1.094	(0.829–1.445)
	rs10889677	A	390 (72.8)	414 (76.7)	1.230	(0.934–1.620)
	rs1343151	C	524 (97.8)	528 (97.8)	1.008	(0.449–2.263)
	rs11209032	A	244 (45.5)	270 (50.0)	1.197	(0.974–1.470)
	rs11805303	T	285 (53.2)	326 (60.4)	1.342	**(1.053–1.709)** **
	rs17375018	A	172 (32.1)	136 (28.2)	0.712	(0.489–1.037)
	rs11209026	A	0	0	–	–
IL17A	rs2275913	A	246 (45.9)	238 (44.1)	0.929	(0.731–1.181)
	rs8193036	T	148 (27.6)	148 (27.4)	0.990	(0.757–1.293)
	rs3804513	T	76 (14.2)	60 (11.1)	0.757	(0.527–1.086)
	rs1974226	T	32 (5.9)	38 (7.0)	1.192	(0.599–2.371)
	rs8193037	A	78 (14.6)	68 (12.6)	0.846	(0.516–1.387)
	rs8193038	C	78 (14.6)	72 (13.3)	0.903	(0.554–1.472)
	rs3748067	A	80 (14.9)	94 (17.4)	1.201	(0.758–1.904)

* *P* = 0.002,

** *P* = 0.017.

+ Patients with UC versus healthy controls.

Note: There were 270 UC patients and 268 healthy controls in this study, so the total number of alleles in one SNP was 270×2 in UC and 268×2 in controls.

### Statistical Analysis

Allele frequencies were first calculated for the Hardy-Weinberg balance test. Genotype, haplotype, and allele frequencies were compared between cases and controls using the chi-square (χ^2^) test, Fisher’s exact test or Student’s t-test, when each was appropriate. Odds radios (OR) and 95% confidence intervals (95% CIs) were applied for 2×2 tables. Bonferroni correction was used for multiple comparisons. Bivariate correlate analysis and binary classification Logistic regression analysis were used to search risk factors associated with SNPs loci mutations. Subgroups were based on clinical and endoscopic severity, disease location, extra-intestinal manifestations, and tobacco smoke history. Subgroups dependent on the relative risk factors were used to find the different distribution of alleles and genotypes.

**Table 3:** The association between IL23R rs17375018/IL17A rs2275913 and Clinical severity of UC.

	OR (95%CI)+			
Variables (n)	IL23R rs17375018		IL17A rs2275913	
	Allele A+	AA+AGvsGG	Allele A+	AA+AGvsGG
Clinical severity				
Mild (106)	0.49*	0.34*	1.05	1.7
	(0.33–0.72)	(0.21–0.54)	(0.77–1.45)	(0.98–2.96)
Moderate (140)	0.88	0.68	0.88	0.71
	(0.64–1.20)	(0.45–1.01)	(0.66–1.18)	(0.46–1.10)
Severe (20)	1.06	0.71	0.45**	0.21***
	(0.49–2.31)	(0.29–1.77)	(0.22–0.91)	(0.82–0.55)

* *P*<0.001,

** *P* = 0.024,

*** *P* = 0.001.

There was 266 subjects included in this comparison, 4 patients with Mayo disease activity index ≤2 were not included.

+ UC Patients versus healthy controls.

Haplotype frequencies and linkage disequilibrium (LD) were estimated and visualized by Haploview 4.2 (http://www.broadinstitute.org//haploview/haploview-downloads). SNPs with minor allele frequencies (MAF) below 5% were excluded. Tagger SNPs were picked up as *r* ^2^ *>*0.8, of which adjacent SNPs with 95% CI = (0.70–0.98) of D’ value were considered the haplotype. Mantel-Haenszel and logistic regression methods were used to determine the interaction between haplotypes in different genes. For all the analyses, differences were considered statistically significant when the *P* value was less than or equal to 0.05.

### Results

Allele frequency of IL23R and IL17A in ulcerative colitis and healthy controls.

Allele frequency data are shown in Table 2 and the allele frequencies met Hardy-Weinberg equilibrium in both UC cases and controls (*P>*0.05; data not shown). The data showed that one disease risk and two protective mutations in the IL23R polymorphisms were found (Table 2). The frequency of the mutant allele T of IL23R rs1884444 and rs7530511 was significantly lower than that of controls (29.6% *vs* 35.4%, *P* = 0.042; 0.7% *vs* 3.3%, *P* = 0.002, respectively). Compared to the controls, the mutant allele T for IL23R rs11805303 was significantly higher in UC patients (60.4% *vs* 53.2%, *P* = 0.0017). Only two (rs7530511 and rs11805303) genome-wide significant SNPs passed the Bonferroni correction threshold (0.05/16 = 0.0031). Our study only showed wild type alleles in IL23R rs11209026 and rs11465804, and heterozygous alleles in IL23R rs11465788 of all the participants. However, we did not find any association between IL17A variant alleles and UC risk.

### Allele or Genotype Frequency of IL23R and IL17A in Subgroups of Ulcerative Colitis

Next, we performed Logistic regression analysis to examine the association of the relative risk factors of these two gene polymorphisms with UC development, and found several significant associations. For example, the IL23R rs17375018 and IL17A rs2275913 variant alleles were associated with UC severity. Allele frequency of IL23R rs17375018 in mild UC was significantly lower than the control subjects (18.9% *vs* 32.1%, *P*<0.001, OR = 0.49, 95%CI = 0.33–0.76, Table 3). The IL17A rs2275913 variant allele frequency distribution in patients with severe UC was significantly different from that in the controls (*P* = 0.024, OR = 0.45, 95% CI = 0.21–0.91, Table 3). Next, we combined the mutant homozygous AA and mutant heterozygous AG compared to the wild homozygous GG, and there were also significant differences for the IL23R rs17375018 in the mild patients (*P*<0.001) and IL7A rs2275913 in the severe patients (*P* = 0.001). From Table 3, we found that the OR value of IL23R rs17375018 was increasing from mild UC to severe UC, but the OR value of IL17A rs2275913 was decreasing. We also compared the average disease activity index in patients who took the variant allele and the wild homozygous patients (Table S2), and the results demonstrated that the IL23R rs17375018 was positively correlated to UC disease severity (AA+AG: 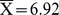; GG: 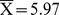; *P* = 0.028), while the IL17A rs2275913 was negatively correlated to UC disease severity (AA+AG: 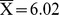; GG: 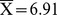; *P* = 0.035). The IL23R rs6677188 genotype distribution between UC endoscopic remission and activity was significantly different (*P = *0.010). The heterozygous type AT frequency distribution was significantly higher in the endoscopic inactivity group than that in the endoscopic activity group (33.8% *vs* 17.2%, *P* = 0.019, OR = 1.958, 95% CI = 1.108–3.460, Table 4). However, no genotype-phenotype association was found between UC disease lesion location or extra-intestinal manifestations and these two (IL23R, IL17A) gene polymorphisms.

**Table 4:** Frequency of IL23R rs6677188 genotypes in UC patients with endoscopic remission and activity.

		Endoscopic staging		
SNP	Genotype	Remission No (%)	Activity No (%)	*P* value+
rs6677188	AT	52 (33.8)	20 (17.2)	**0.019**
	AA	6 (3.9)	6 (5.17)	0.630
	TT	96 (62.3)	90 (77.6)	0.253

Endoscopic stage was assessed by Rachmilewitz endoscopic index, Endoscopic remission**:** 0–4, Endoscopic activity: 5–12.

+ Endoscopic remission group versus Endoscopic activity group.

**Figure 1:**
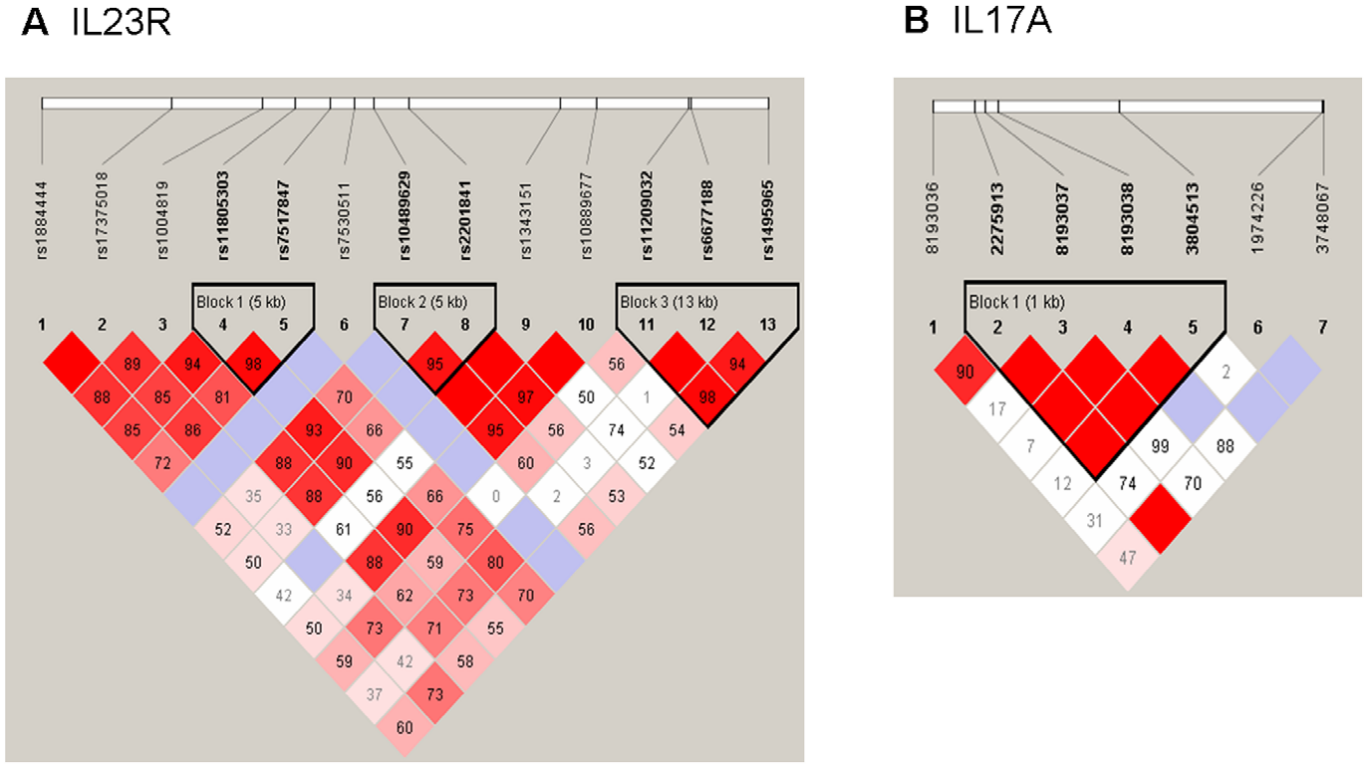
Linkage disequilibrium patterns of the region around the IL23R and 17A gene in the Chinese Han population. Numbers indicate the extent of D’ between 2 SNPs and in the dark area which have no digital respective D’ = 1. Dark color indicates strong connection. Three linkage disequilibrium blocks were found in IL23R, and one LD block was in IL17A. A, LD patterns of IL23R. B, LD patterns of IL17A.

### Haplotypes of IL23R and IL17A and Gene-gene Interaction

Then, we measured the linkage disequilibrium (LD) patterns of the SNPs and identified three LD blocks from the SNPs of IL23R and one LD block in the SNPs of IL17A (Figure 1). To determine whether any specific haplotype would confer a higher risk or protection for UC, we performed specific and global-haplotype tests of association. Analysis of the haplotype structure revealed 15 distinct haplotypes, and 4 of them were associated with UC (Table 5). The most common haplotype (TT) of IL23R, formed with rs11805303 and rs7517847, was observed to be a risk haplotype, with a frequency of 60% in UC patients and 50% in controls (*P* = 0.020). The rare haplotype (GGTT) of IL17A, formed with four SNPs (rs2275913, rs8193037, rs8193038, and rs3804513), was also a risk haplotype with a frequency of 3.3% in UC patients and 0.7% in controls (*P* = 0.034). Another two haplotypes of IL23R were both protective factors, one was tagged by the rs10489629-A and rs2201841-T alleles, and the frequency of this haplotype was 0.7% in UC patients and 3.7% in controls (*P* = 0.019). Another rare haplotype formed with three wild type alleles (GTG) of rs11209032, rs6677188 and rs1495965, the frequency of which was 1.1% in UC patients and 4.1% in controls (*P* = 0.029).

**Table 5:** Frequency of IL23R and IL17A haplotypes in UC patients and healthy controls.

Gene							FrequencyUC withHaplotypePresent	FrequencyControls withHaplotypePresent	*P* value+
**IL23R**			rs11805303	rs7517847					
		H1	C	G			0.356	0.418	0.138
		H2	C	T			0.041	0.078	0.062
	risk	H3	T	T			0.600	0.500	**0.020**
			rs10489629	rs2201841					
		H1	A	C			0.756	0.709	0.222
	protective	H2	A	T			0.070	0.370	**0.019**
		H3	G	T			0.226	0.250	0.624
		H4	G	C			0.011	0.004	0.521
			rs11209032	rs6677188	rs1495965				
		H1	A	T	G		0.496	0.455	0.340
		H2	G	A	A		0.170	0.213	0.257
		H3	G	T	A		0.315	0.291	0.612
	protective	H4	G	T	G		0.110	0.410	**0.029**
**IL17A**			rs2275913	rs8193037	rs8193038	rs3804513			
		H1	A	G	T	A	0.443	0.457	0.744
		H2	G	G	T	A	0.397	0.391	0.883
		H3	G	A	C	T	0.126	0.137	0.717
	risk	H4	G	G	T	T	0.033	0.007	**0.034**

+ Patients with UC versus healthy controls.

**Figure 2:**
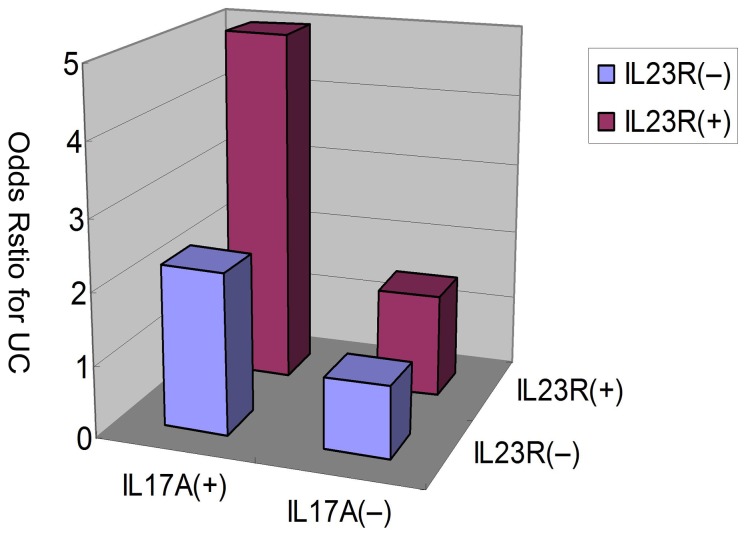
Increase of ulcerative colitis risk with the haplotypes of IL23R and IL17A. Relative risk haplotypes were plotted and demonstrate association between IL23R block1 haplotype3 and IL17A haplotype4 (p = 0.014 for interaction).

In the analysis of gene-gene interactions of the IL23/IL17 genes, we combined the two risk haplotypes of IL23R and IL17A together, and found a significant increase in UC risk in IL23R block1 Haplotyp3, when the risk haplotype of IL17A, Haplotyp4, was also present (*P* = 0.004 by Mantel-Haenszel test, Table 6). The interaction between these two risk haplotypes was statistically significant (*P* = 0.014). Furthermore, the “risk” haplotypes of IL23R and IL17A synergistically increased UC risk (Figure 2), suggesting that there was a gene-gene interaction between the IL23R and IL17A haplotypes in the IL-23/IL-17 pathway that affected the pathogenesis of UC.

**Table 6:** Interaction between IL23R risk haplotype and IL17A risk haplotype in all UC patients.

Presence of IL23R Block1 H3	Presence ofIL17A H4	UC	Control	OR	0.95% Confidence Interval	Mantel-Haenszel*P* value	Interaction *P* value
−	−	216	268	1			
−	+	2	0	2.24	2.03–2.48		
+	−	306	264	1.44	1.13–1.83	**0.004**	**0.014**
+	+	16	4	4.96	1.64–15.06		

H3: Haplotype 3; H4: Haplotype 4.

## Discussion

To date, there is emerging evidence showing that the IL-23/IL-17 axis plays an important role in UC development [10], [26]. In this regard, recent studies have suggested that Th17 cells mediated intestinal inflammation [27]–[28]. IL-23 was reported to promote production of IL-17 and to be required for Th17 differentiation in a pro-inflammatory context [29]–[31], especially in the presence of TGF-β and IL-6 for production of IL-17A, IL-17F, IL-6, IL-22, IL-26, TNF-α and GM-CSF [30], [32]. IL-23 and Th17 cytokines act coordinately to influence the balance between tolerance and immunity in the intestine [33]. Moreover, a study on linking genetic susceptibility to CD with Th17 cell function demonstrated that IL23R genotypes influence Th17 cytokine IL-22 serum expression [5]. IL-22 is considered to have protective functions in IBD in many studies [34]–[35]. Genetic studies showed that IL23R variants were associated with both CD and UC [36]. Recently, an accumulating body of evidence indicated that the IL23R genotype might affect the response to anti-TNF therapy in UC [37].

In our study, the data were the first findings to show that the IL23R rs7530511 and rs11805303 variants were associated with UC susceptibility in a Chinese Han population. IL23R rs7530511 variant allele was protective factor for UC, while rs11805303 was significantly associated with an increased risk for UC development. We also demonstrated that patients with the IL23R haplotypes who also carry the IL17A risk haplotypes had higher susceptibility to UC. This indicates that gene-gene interaction between IL23R and IL17A might occur.

A study from Magyaril *et al*. [17] showed that the IL23R gene variants, rs10889677 C/A and rs2201841 T/C appeared to increase susceptibility to CD. However, the rs10889677 and rs2201841 in our study had no significant relationship with UC susceptibility. Ferguson *et al*. [38] detected five common SNPs in IL23R gene (rs11805303, rs7517847, rs1343151, rs11209026, and rs10889677) in CD patients from a New Zealand population and found that rs1343151 and/or rs7517847 variants strongly reduced the risk of developing CD at both allelic and genotype levels. Nevertheless, we did not find the apparent association between these SNPs in IL23R with UC susceptibility, but the rs11805303 and rs7517847 could form three haplotypes with TT to increase UC susceptibility. The IL23R rs11209026 was reported to be associated with IBD and many other immune-related diseases [39]–[44]. Several studies reported that the rs11209026 variant allele A was significant lower in CD patients than the controls; this indicated that the IL23R rs11209026 variant confers a protective effect against CD [39]–[41], [45]. However, the association between rs11209026 variant and UC susceptibility has some controversy. Some studies revealed that there was no relationship between rs11209026 and UC [39]–[40], but other studies have demonstrated that the IL23R rs11209026 variant also conferred a protective effect against UC [44]–[47]. Glas *et al*. [47] analyzed 10 IL23R SNPs (including 9 SNPs in our study) in a large German IBD cohort, and found that the rs1004819 was the major IL23R variant associated with CD in the German population, while the rs11209026 IL23R variant was a protective marker for CD and UC. Furthermore, all IL23R gene variants in this study showed highly significant associations with CD, and 7 IL23R SNPs variants were significantly associated with UC except rs11465804 and rs11209032. However, in our study, there was only wild type allele G on IL23R rs11209026 in UC and controls. We did not find any association of these IL23R gene polymorphisms with UC.

IL-17 is associated with host defense against infectious agents and chronic inflammation [28]. IL17A is located on 6p12.1, a genomic region containing putative susceptibility loci (IBD3) for IBD. Moreover, a few studies have recently reported the associations between the G-197A in the IL7A promoter and the UC phenotype. Arisawa *et al*. [48] showed a significant association between polymorphisms of IL17A and IL17F genes and UC. Both IL17A (rs2275913, G-197A) and IL17F (rs763780, 7488T) alleles were significantly associated with the increased risk in the development of UC. However, our data did not support this. The IL17A (rs2275913, G-197A) variant allele did not associate with UC susceptibility in our patients. But in a subgroup analysis, we found that the IL17A (rs2275913, G-197A) variant was associated with disease severity; patients with mutant allele gene A tended towards mild disease severity.

From the LD analysis, we found that three haplotypes of IL23R and one haplotype of IL17A were significant associated with UC in the Chinese Han population. The haplotypes AT and GTG from the IL23R block2 H2 and block3 H4 were two protective haplotypes that reduced UC susceptibility. In contrast, the haplotypes TT and GGTT from IL23R and IL17A, respectively, were two risk haplotypes that increased UC susceptibility. These data are novel and have not been reported before. Einarsdottir *et al.* [16] showed that two haplotypes TTCTGAAA and CTCTGCAA (rs1004819, rs10489629, rs2201841, rs11465804, rs11209026, rs1343151, rs10889677, and rs11209032) were associated with UC risk, but a report from Lappalainen *et al.* [15] showed that the haplotype CTCTGCAA had no association with UC risk.

In addition, we demonstrated the most important finding of the gene-gene interaction between IL23R and IL17A that could increase the risk of UC susceptibility in the Chinese people. The data demonstrated that there was an increased odds ratio for UC as the “risk” haplotypes for the two genes were combined. While the OR for each “risk” haplotype alone was relatively modest, the combined OR for the identified haplotypes was substantially greater than 4. This finding suggested that there was a gene dosage effect of the IL23R and other genetic variants within IL17A in the IL-23/IL-17 pathway in increased UC risk. McGovern *et al.* [49] found some “risk” and “protective” haplotypes of IL23R and IL17A in CD patients. Another study from Kim *et al.* [18] showed significant increases of UC risk when decreasing protective or increasing risk alleles. Consequently, cumulative risk alleles of IL17A diminished the protective OR of IL23R in IBD patients, demonstrating an additive risk of UC along with an increase in the number of risk alleles in IL17A. These results provide insights into the genetic interactions in the IL23/IL17 pathway.

Finally, in our study, we also found an association of these two gene SNPs with disease severity and other clinical characteristics in UC. The variant allele A for the IL23R rs17375018 was positively correlated to UC disease severity, but the IL17A rs2275913 variant allele A had a negative relationship with UC disease severity. In IL23R rs6677188 patients with heterozygous type AT had a higher endoscopic remission rate. However, Tremelling *et al*. [50] analyzed eight IL23R SNPs including rs1343151 and found that there was no significant genotype-phenotype correlation based on sub-groups of UC. No genotype-phenotype association was found in the lesion localizations and extra intestinal manifestations of UC in our study.

In summary, our study investigated the association of SNPs of IL23R and IL17A with UC risk in Chinese Han population. The most novel finding was the linkage of the IL23R and IL17A gene regions to UC and the gene-gene interactions in the IL-23/IL-17 pathway that could increase the risk of UC susceptibility in this Chinese population. However, our study also has some limitations, such as no extensive coverage of both genes, and small size of the cohort. Thus, future study will confirm our current data using a more diverse and larger UC population.

## Supporting Information

Table S1 The SNP positions of IL23R and IL17A.
(DOC)

Table S2 The association between IL23R rs17375018/IL17A rs2275913 and Clinical severity of UC (Student’s t-test).
(DOC)
