# Supplementary material for: Association of Single Nucleotide Polymorphisms of IL23R and IL17 with Ulcerative Colitis Risk in a Chinese Han Population
Source: PLoS One. 2012 Sep 11;7(9):e44380. doi: 10.1371/journal.pone.0044380 (PMC3439435; doi:10.1371/journal.pone.0044380)
Supplement: Table S1 — The SNP positions of IL23R and IL17A. (DOC) [file pone.0044380.s001.doc]

**Table S1 The SNP positions of IL23R and IL17A**

| **SNP** | | **Chromosome** | **Position** |
| --- | --- | --- | --- |
| **IL23R** | rs1004819 | 1 | 37,642,131 |
|  | rs1495965 | 1 | 37,725,426 |
|  | rs1884444 | 1 | 37,605,730 |
|  | rs2201841 | 1 | 37,666,120 |
|  | rs6677188 | 1 | 37,712,321 |
|  | rs7517847 | 1 | 37,653,587 |
|  | rs7530511 | 1 | 37,657,305 |
|  | rs10489629 | 1 | 37,660,267 |
|  | rs10889677 | 1 | 37,697,038 |
|  | rs1343151 | 1 | 37,691,047 |
|  | rs11209032 | 1 | 37,712,010 |
|  | rs11805303 | 1 | 37,647,434 |
|  | rs17375018 | 1 | 37,627,065 |
|  | rs11209026 | 1 | 37,677,876 |
|  | rs11465804 | 1 | 37,674,444 |
|  | rs11465788 | 1 | 37,620,212 |
| **IL17A** | rs2275913 | 6 | 51,991,033 |
|  | rs8193036 | 6 | 51,990,493 |
|  | rs3804513 | 6 | 51,993,197 |
|  | rs1974226 | 6 | 51,995,335 |
|  | rs8193037 | 6 | 51,991,109 |
|  | rs8193038 | 6 | 51,991,382 |
|  | rs3748067 | 6 | 51,995,339 |
