# Supplementary material for: Association of Single Nucleotide Polymorphisms of IL23R and IL17 with Ulcerative Colitis Risk in a Chinese Han Population
Source: PLoS One. 2012 Sep 11;7(9):e44380. doi: 10.1371/journal.pone.0044380 (PMC3439435; doi:10.1371/journal.pone.0044380)
Supplement: Table S2 — The association between IL23R rs17375018/IL17A rs2275913 and Clinical severity of UC (Student’s t-test). (DOC) [file pone.0044380.s002.doc]

**Table S2 The association between IL23R rs17375018/IL17A rs2275913 and Clinical severity of UC (Student’s t-test).**

|  |  | **Genotypes** | |  |
| --- | --- | --- | --- | --- |
| **SNP** |  | **AA+AG** | **GG** | ***P* value** |
| rs17375018 | No (%) | 112 (42.1) | 154 (57.9) |  |
|  | Mean UCAI | 6.91 | 5.97 | **0.028** |
| rs2275913 | No (%) | 184 (69.2) | 82 (30.8) |  |
|  | Mean UCAI | 6.02 | 7.04 | **0.035** |

UCAI ulcerative colitis activity index
